# Plasmablastic Lymphoma of the Anorectal Region in a Human Immunodeficiency Virus (HIV)-Positive Patient: Integrating Radiologic Findings

**DOI:** 10.7759/cureus.90741

**Published:** 2025-08-22

**Authors:** Erwin J Narváez-García, Aldo M Pacheco-Carrillo, Flor E Ortiz-Villeda, Matías Salinas-Chapa, Guillermo Elizondo-Riojas, Rodolfo Franco-Marquez

**Affiliations:** 1 Radiology, Hospital Universitario "Dr. José Eleuterio González", Monterrey, MEX; 2 Pathology, Hospital Universitario "Dr. José Eleuterio González", Monterrey, MEX

**Keywords:** aids-related malignancies, anal lymphoma, anal tumor, extraoral plasmablastic lymphoma, plasmablastic lymphoma, radiologic features, radiologic findings

## Abstract

Plasmablastic lymphoma (PBL) is an uncommon and aggressive subtype of diffuse large B-cell lymphoma (DLBCL), strongly linked to immunosuppressed states. Although it most frequently involves the oral cavity, anorectal presentation is exceptionally rare. We describe the case of a 27-year-old male with a recent human immunodeficiency virus (HIV) diagnosis, who presented with a growing perianal mass and rectal bleeding. The lesion progressed despite initial treatment and required surgical management. Imaging revealed a necrotic anal canal mass with soft tissue invasion, and histopathology confirmed the diagnosis of PBL. The patient underwent chemotherapy but ultimately experienced a poor clinical outcome. Due to its rarity and non-specific presentation, anorectal PBL is often misdiagnosed, delaying definitive treatment. Imaging, particularly magnetic resonance imaging (MRI), is essential for evaluating lesion extent and guiding clinical decisions. Despite available therapies, the prognosis remains guarded. This case highlights the importance of considering PBL in the differential diagnosis of atypical anorectal masses in immunosuppressed patients. Early imaging and histopathologic assessment are vital for timely diagnosis and treatment.

## Introduction

Plasmablastic lymphoma (PBL) is a rare and highly aggressive subtype of diffuse large B-cell lymphoma (DLBCL) [1]. Since its initial description by Delecluse et al. in 1997, PBL has remained a diagnostic and therapeutic challenge. It represents an uncommon neoplasm, accounting for approximately 2.6% of acquired immunodeficiency syndrome (AIDS)-related lymphomas [2]. 

Although its precise etiology remains unclear, a strong association with human immunodeficiency virus (HIV) infection has been consistently observed. In the original case series by Delecluse et al., 15 out of 16 patients were HIV-positive [2,3]. Subsequent studies have confirmed this relationship, with estimates suggesting that up to two-thirds of reported cases occur in HIV-positive individuals. However, PBL has also been documented in patients with other forms of immunosuppression, including solid organ transplantation and, less commonly, in immunocompetent hosts [4].

The pathophysiology of PBL is not fully understood, but it is thought to originate from plasmablast-activated B cells that have undergone somatic hypermutation and class-switch recombination but have not yet terminally differentiated into plasma cells. Most HIV-associated lymphoproliferative disorders - including primary central nervous system lymphoma, systemic DLBCL IB-plasmacytoid, primary effusion lymphoma (PEL) and its solid variant, and oral cavity PBL - share a plasmacytic phenotype and are frequently associated with Epstein-Barr virus (EBV) infection [5].

The most common anatomical site of PBL is the oral cavity. However, less frequent extraoral presentations have been described in the skin, gastrointestinal tract, lungs, maxillary sinuses, bone, lymph nodes, and anal canal. Involvement of the anal canal is exceedingly rare, with only a limited number of cases reported in the literature [6].

We report the case of a 27-year-old male with a recent diagnosis of HIV, who presented with anal canal PBL. We aim to contribute to the limited literature on this rare presentation by highlighting its clinical course and radiologic characteristics.

## Case presentation

A 27-year-old male with a recent diagnosis of HIV infection presented in April 2024 with a 1 cm perianal mass and hematochezia. Over the following weeks, the lesion increased in size, reaching approximately 5 cm, and was accompanied by intermittent low-grade fevers and purulent discharge. Initial outpatient management with oral antibiotics was prescribed, but there was no clinical improvement.

**Figure 1:**
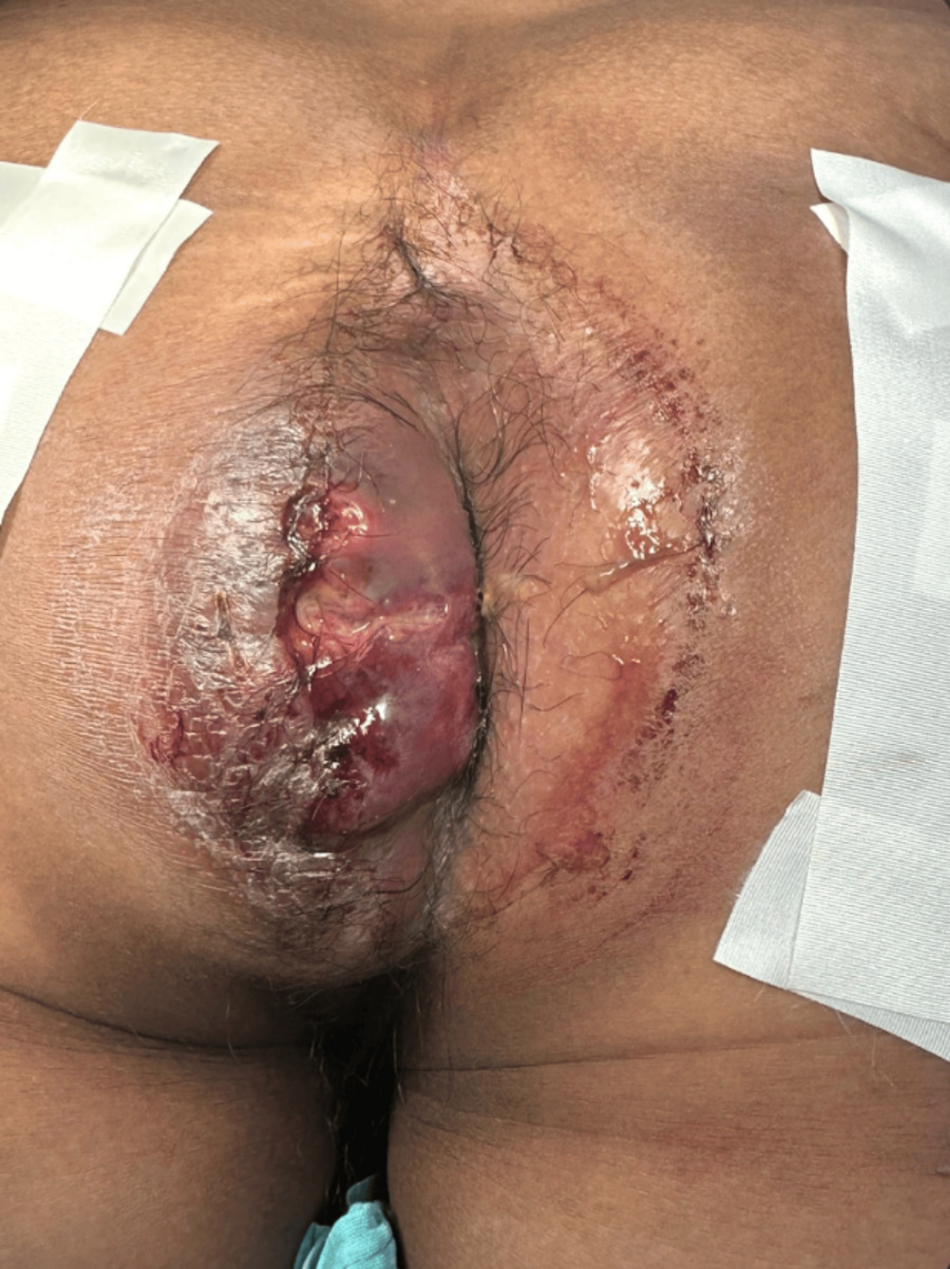
Clinical image Suppurative perianal lesion located at 9 o’clock from the anal verge that causes mass effect on the gluteal fold.

He was evaluated at our institution on May 28, 2024, and admitted with a presumptive diagnosis of a suppurative perianal mass at 9 o’clock from the anal verge (Figure 1). On arrival, his CD4 lymphocyte count was 200 cells/mm³, and his viral load was undetectable. Anoscopy with biopsy was performed, and the patient was discharged for outpatient follow-up with medical treatment. However, on July 6, 2024, he was readmitted to the Emergency Department due to syncope, decreased level of consciousness, and persistent hematochezia for the previous two weeks. On examination, the perianal mass had significantly enlarged and was actively bleeding, with hemodynamic instability. Surgical debridement, lavage, and exploratory laparotomy with colostomy were performed. Broad-spectrum antibiotics and blood transfusions were administered. 

**Figure 2:**
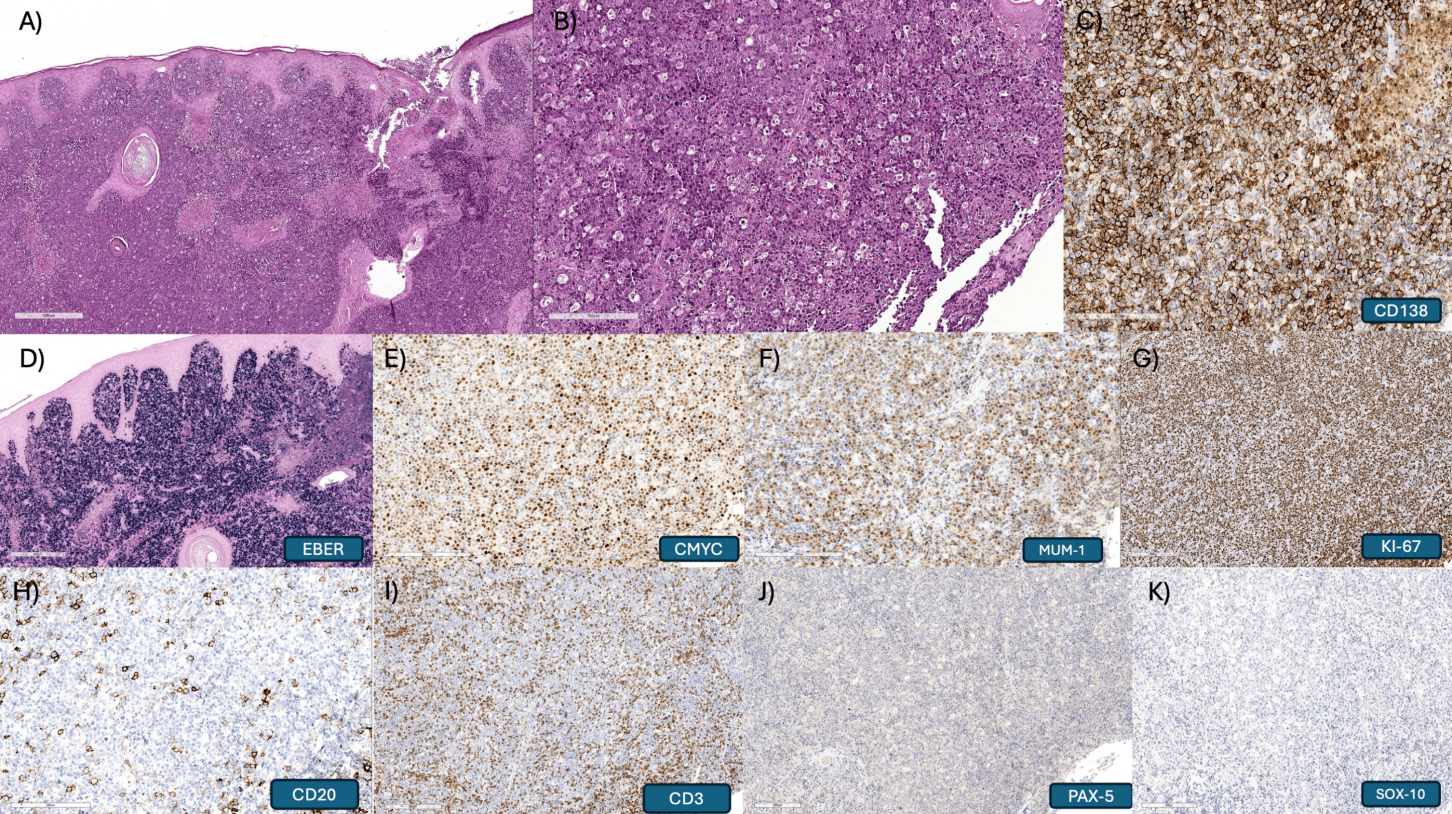
Histopathology (A) At low magnification, the lesion displays a diffuse pattern underlying the epithelium (hematoxylin and eosin). (B) It is composed of sheets of intermediate- to large-sized lymphoid cells exhibiting immunoblastic and plasmablastic morphology, accompanied by numerous tingible body macrophages and scattered apoptotic bodies (hematoxylin and eosin). Immunohistochemical staining demonstrated positivity for CD138 and MUM1 (C, D), supporting plasmablastic differentiation. Expression of C-MYC and positivity for Epstein-Barr virus-encoded RNA by in situ hybridization (EBER-ISH) further support the diagnosis of plasmablastic lymphoma (E, F). The Ki-67 proliferation index was markedly elevated, exceeding 90% (G). The neoplastic cells were negative for CD20, CD3, PAX-5, and SOX-10 (H-K).

Histopathological analysis confirmed the diagnosis of PBL, with immunohistochemistry positive for CD138, MUM1, and a Ki-67 proliferation index elevated above 90%; there was also positive expression for C-MYC and Epstein-Barr virus-encoded RNA by in situ hybridization (EBER-ISH) (Figure 2). 

**Figure 3:**
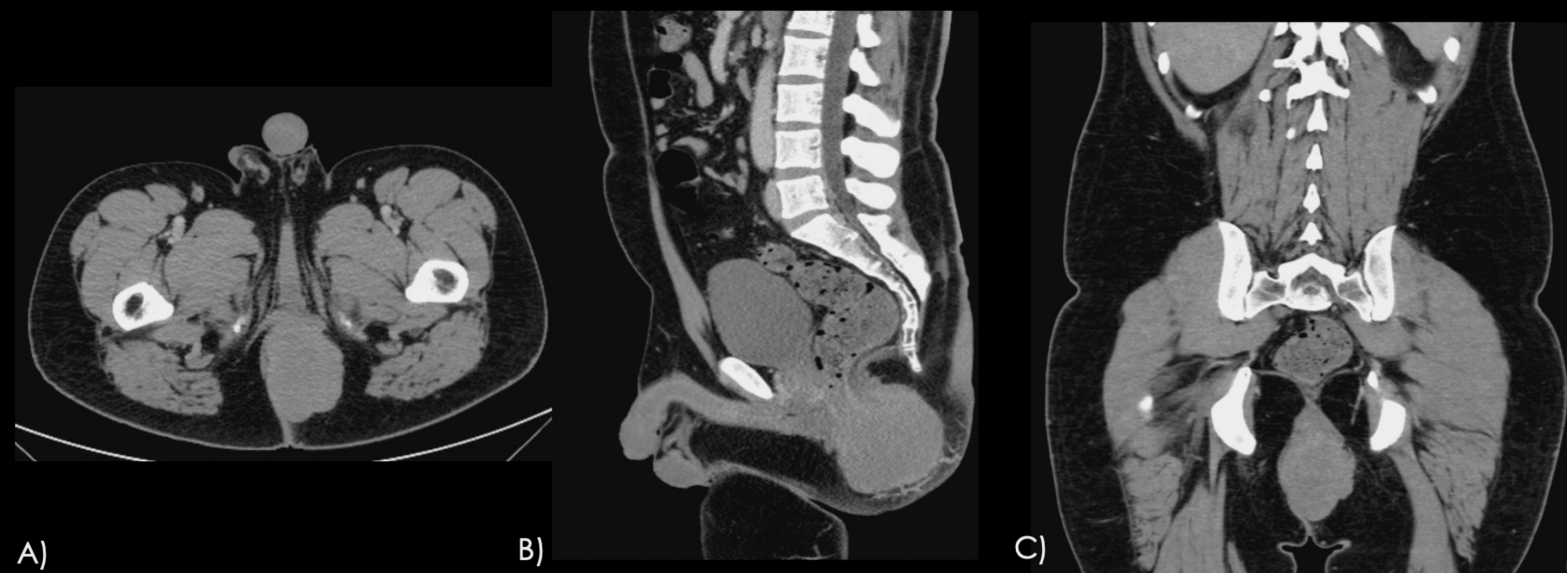
Baseline contrast-enhanced CT Baseline contrast-enhanced CT scan in the venous phase: (A) axial, (B) sagittal, and (C) coronal sections. A well-defined, lobulated mass with slight homogeneous contrast uptake, centered in the anal canal, causing rightward deformation of the gluteal fold. CT, computed tomography

**Figure 4:**
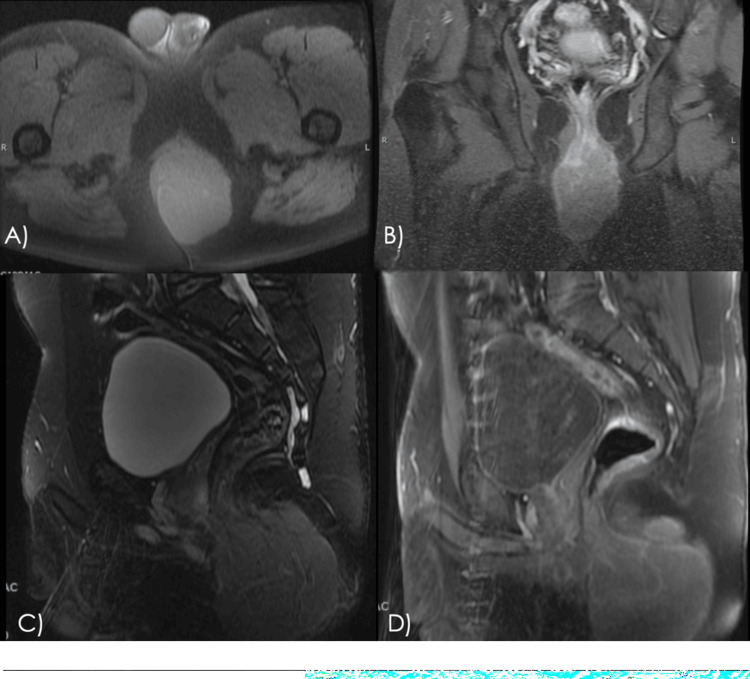
Baseline contrast-enhanced MRI Pelvic contrast-enhanced MRI. T1-weighted axial (A) and coronal post-gadolinium T1-weighted (B) images, both with fat saturation. The coronal image shows tumoral infiltration of the internal anal sphincter. T2-weighted sagittal (C) and post-gadolinium T1-weighted sagittal (D) images, both with fat saturation. A lobulated mass involves the anal canal and shows heterogeneous enhancement. MRI, magnetic resonance imaging

A contrast-enhanced computed tomography (CT) scan of the abdomen on July 8, 2024, revealed an irregular perianal mass with ill-defined borders, peripheral enhancement, and a central hypodense area suggestive of necrosis (Figure 3). The lesion measured approximately 9.6 × 9.3 × 5.6 cm and involved the corpus cavernosum and spongiosum of the penis, as well as the levator ani muscle. Mucosal thickening of the anorectal region was also noted, suggestive of a neoplastic process. A contrast-enhanced magnetic resonance imaging (MRI) study showed a well-defined, lobulated solid lesion originating from the left lateral wall of the distal anal canal. The mass measured 9.0 × 7.5 × 5.1 cm, appeared isointense on T1- and hyperintense on T2-weighted sequences, and demonstrated peripheral and heterogeneous slight enhancement. It extended into the left ischioanal fossa, displaced the anal canal to the right, and infiltrated the external anal sphincter (Figure 4). 

**Figure 5:**
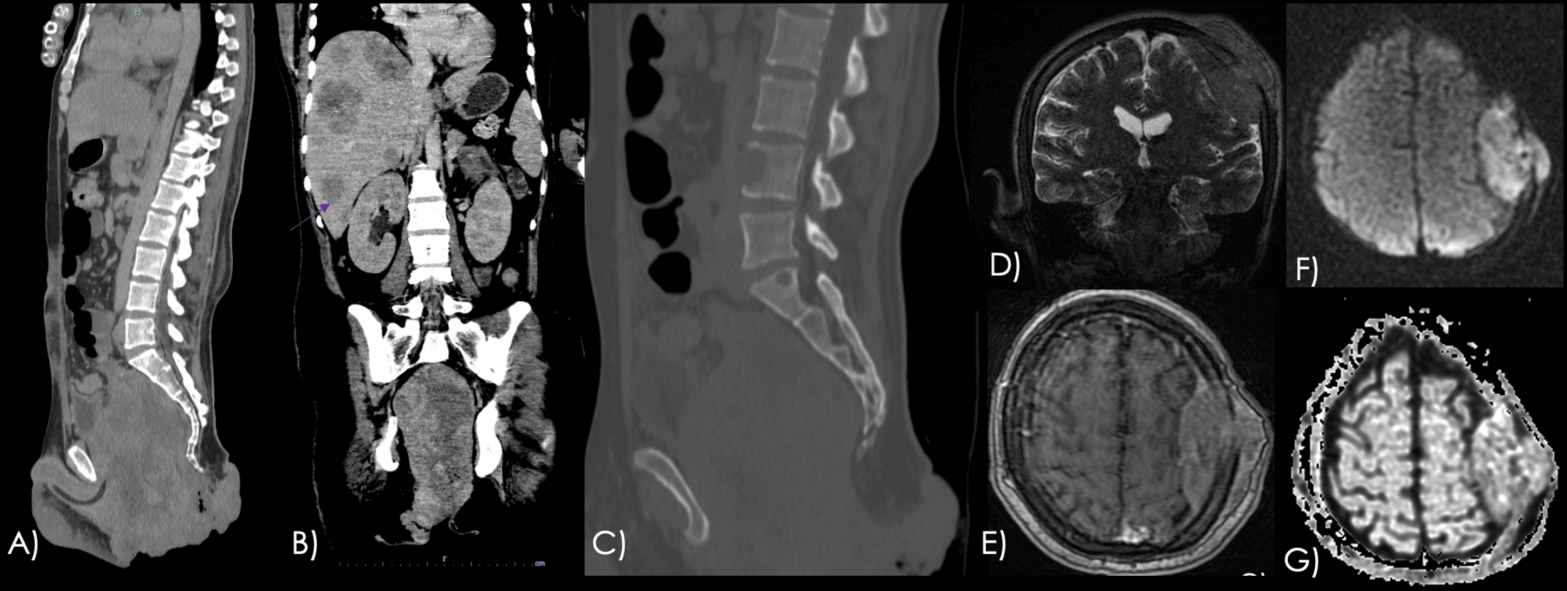
Follow-up imaging showing recurrence and disease progression Contrast-enhanced CT abdominal scan. A large, irregular, and infiltrative mass is identified, involving the entire pelvic cavity, infiltrating the corpora cavernosa, and displacing the bladder (A). Multiple rounded lesions with diffuse distribution and heterogeneous appearance are seen, consistent with metastatic disease (B). In the bone window, lytic lesions are identified in multiple lumbar vertebral bodies (C). Contrast-enhanced brain MRI T2-weighted coronal (D), T1-weighted axial post-gadolinium (E), axial DWI (F), and ADC map (G) images show an extra-axial supratentorial lesion in the left frontoparietal region, demonstrating homogeneous contrast enhancement. The lesion involves the calvarium, originates from the meninges, and shows marked diffusion restriction. The corresponding ADC value was measured at 0.67 × 10^-3^ mm^2^/s, consistent with true diffusion restriction, in keeping with lymphoma. CT, computed tomography; MRI, magnetic resonance imaging; DWI, diffusion-weighted imaging; ADC, apparent diffusion coefficient

He started a chemotherapy regimen based on rituximab, cyclophosphamide, doxorubicin, vincristine, prednisone (R-CHOP), and bortezomib. Four months later, the patient was readmitted with recurrent severe rectal bleeding. Imaging studies at that time revealed local recurrence of the lesion, along with hepatic, osseous, and meningeal metastases (Figure 5). Despite the initiation of systemic chemotherapy and antiretroviral therapy, the patient’s condition deteriorated with unresponsive hypovolemic shock, and he ultimately died from disease progression.

## Discussion

Non-Hodgkin lymphoma (NHL) is the second most common malignancy among patients with HIV infection. HIV increases the overall risk of lymphoma by 1%-6% per year, and individuals with AIDS have an approximately 60-fold higher risk of developing lymphoma compared to the general population [5]. DLBCL is the most frequent subtype, accounting for approximately 41% of HIV-associated NHL cases [7].

Although it has a strong association with HIV, a growing number of cases have been reported in patients with other causes of immunosuppression, including solid organ transplantation. EBV co-infection is also frequently observed, with up to 60% of PBL cases showing EBV positivity [3,8]. 

The most commonly affected demographic is males in their fourth to fifth decades of life [9,10]. While the oral cavity remains the most frequent site of involvement, extraoral presentations have been described in the paranasal sinuses, gastrointestinal tract, skin, and soft tissues [4,8,9,11]. Anal canal involvement is exceedingly rare, representing only 0.1% of all anal malignancies and being one of the least common anatomical locations described in the literature for PBL [12].

In our case, the patient's symptoms aligned with previously documented patterns, presenting as a tender perianal mass with associated bleeding. Notably, his age was well below the average reported in similar cases. In advanced stages, the disease can become a medical emergency, either due to hypovolemia from severe hematochezia or bowel obstruction secondary to mass effect.

Imaging studies play a critical role in the initial evaluation and staging of the disease. CT is often the first imaging modality used due to its widespread availability and cost-effectiveness in the workup of lymphomas. In our case, CT findings mirrored those reported in previous case reports, revealing a heterogeneous mass with mild contrast enhancement and central necrosis, extending into adjacent soft tissues [8-10]. 

MRI, however, remains the gold standard for assessing anal canal PBL, owing to its superior spatial resolution and ability to provide detailed lesion characterization. MRI findings typically reveal a rapidly growing lobulated mass, heterogeneous in signal intensity, often hypointense on T1-weighted images, with mild gadolinium enhancement and evidence of local tissue infiltration. In our case, there was notable involvement of the corpora cavernosa, although other reports have documented extension into surrounding structures such as the seminal vesicles, rectal wall, and, in women, the vagina [10]. 

In our case, no lymphadenopathy was identified in any nodal chains in the abdomen or thorax. However, previous reports have demonstrated nodal involvement on staging scans, including lymph node infiltration both above and below the diaphragm [10,11]. Distant metastases in our patient emerged abruptly, highlighting the aggressive biological behavior of this neoplasm. In a similar case, Rivera and Baalwa described a patient with PBL who developed pleural involvement with effusion, as well as cerebral and probable renal metastases [13]. 

Chemotherapy remains the cornerstone of PBL management. However, its clinical course is typically aggressive and prone to early relapse, contributing to poor overall outcomes even when intensive multi-agent chemotherapy or autologous stem cell transplantation is employed.

Multiple chemotherapy regimens have been used in the treatment of PBL, including CHOP, Hyper-CVAD/MA (hyperfractionated cyclophosphamide, vincristine, doxorubicin, dexamethasone, high-dose methotrexate, and cytarabine), CODOX-M/IVAC (cyclophosphamide, vincristine, doxorubicin, high-dose methotrexate/ifosfamide, etoposide, and high-dose cytarabine), COMB (cyclophosphamide, vincristine, methyl-CCNU, and bleomycin), and dose-adjusted EPOCH (etoposide, prednisone, vincristine, cyclophosphamide, and doxorubicin) [4]. No single regimen has shown clear superiority, and treatment is often tailored based on patient comorbidities, performance status, and institutional experience.

This malignancy is highly aggressive and characterized by rapid progression with a high mortality rate. As observed in our case, the prognosis of PBL remains poor, with most patients succumbing to the disease within the first year following diagnosis [8]. In this case, timely recognition of relevant clinical factors, such as immunosuppression, in conjunction with radiologic and histopathological evaluation, was essential for establishing the diagnosis. Although therapeutic options remain limited, early detection may offer a window of opportunity for potentially curative intervention [9]. 

Although an exact statistic regarding the proportion of PBL cases involving the anal canal is not available, our review of the literature suggests that fewer than 50 cases have been reported worldwide. This underscores the rarity of this presentation and highlights an opportunity for further investigation and systematic reviews.

## Conclusions

Anal canal involvement by PBL represents a highly aggressive manifestation of this rare neoplasm, typically occurring in the context of immunosuppression. Our case highlights the critical role of imaging - particularly MRI - in the early detection, anatomical mapping, and treatment planning of this entity. Given the overlap in clinical features with other anorectal pathologies, the standardization and recognition of characteristic radiologic patterns are essential for timely diagnosis. A unified radiologic approach may improve diagnostic accuracy and contribute to earlier therapeutic intervention in this highly lethal disease.
